# Safety Evaluation of Topical Application of Nano-Liposomal Form of Amphotericin B (SinaAmpholeish) on Healthy Volunteers: Phase I Clinical Trial

**Published:** 2019

**Authors:** Seyed Ebrahim ESKANDARI, Alireza FIROOZ, Mansour NASSIRI-KASHANI, Mahmoud Reza JAAFARI, Amir JAVADI, Akram MIRAMIN MOHAMMADI, Ali KHAMESIPOUR

**Affiliations:** 1. Center for Research and Training in Skin Diseases and Leprosy, Tehran University of Medical Sciences, Tehran, Iran; 2. Nanotechnology Research Center, Pharmaceutical Technology Institute, Mashhad University of Medical Sciences, Mashhad, Iran; 3. Department of Pharmaceutical Nanotechnology, School of Pharmacy, Mashhad University of Medical Sciences, Mashhad, Iran; 4. Department of Social Sciences, School of Medicine, Qazvin University of Medical Sciences, Qazvin, Iran

**Keywords:** Safety, Topical formulation, Nano-liposomal amphotericin B, Cutaneous leishmaniasis

## Abstract

**Background:**

We aimed to evaluate the safety of SinaAmpholeish in a double-blind, randomized, phase 1 clinical trial in healthy human volunteers.

**Methods:**

The study was carried out in DermaLab of Center for Research and Training in Skin Diseases and Leprosy, Tehran University of Medical Sciences, Tehran, Iran in 2012. A topical Nano-liposomal formulation of 0.4% Amphotericin B was developed against *Leishmania* under trade name of SinaAmpholeish. In this randomized, double-blind, right-left, comparative, phase I clinical trial, in 2 steps; 7 and 20 healthy volunteers were recruited and applied SinaAmpholeish on the right and its vehicle on the left volar side of forearm, twice a day for one week or 3 times a day for two weeks. Seven biophysical skin parameters were measured in standard conditions before and 2 wk after application.

**Results:**

There was no adverse effect when SinaAmpholeish and its vehicle were used twice a day for seven days. However, when were used 3 times a day for two weeks, both SinaAmpholeish and its vehicle induced severe local skin reactions in 2 volunteers leading to discontinuation of application. Mild and temporary local reactions were observed in about half of the application sides and there was no significant difference between SinaAmpholeish and its vehicle.

**Conclusion:**

The new formulation is safe and worth to be tested in further phase 2 clinical trial and since there was no adverse effect with twice a day application it was decided to use SinaAmpholeish twice a day in phase 2 clinical trial.

## Introduction

*C*utaneous leishmaniasis (CL) is endemic in more than 80 countries, 70 of them developing ones. Iran is among 7 countries where 90% of all cases of CL occur. CL, either zoonotic (ZCL) caused by *L. major* or anthroponotic (ACL) caused by *L. tropica,* is endemic in 17 of 31 provinces of Iran (1–4). CL patients are even seen in non-endemic cities due to travel to endemic areas (5).

Although CL is a self-limiting lesion, healing process is slow. It might take up to years, and leaving scars. Various treatment modalities have been used in the treatment of CL, but still, the standard treatment of CL is antimoniate derivatives [meglumine antimoniate (Glucantime®) and sodium stibogluconate (Pentostam®)]. The standard treatment has to be administered as multiple systemic or intralesional injections, associated with severe side effects and significant discomfort. A published systematic review on the treatment of OWCL demonstrated that no effective, safe and inexpensive treatment is currently available for CL. Therefore, search for more effective, safe, and affordable treatment for CL is inevitable. Various drugs have been studied for the treatment of CL, but those reached to phase 3 clinical trial did not show enough efficacy (6–13).

Amphotericin B and its liposomal formulation have been used successfully for the treatment of visceral leishmaniasis (VL), extensive and complicated forms of CL, but they are toxic and expensive and require prolonged infusion (11–12). A new topical liposomal formulation of amphotericin B (topical Nano-liposomal amphotericin B 0.4%, with the trade name of SinaAmpholeish) has been developed in Mashhad University of Medical Sciences in Iran (14). The formulation was shown to be effective against *L. major* and *L. tropica* in vitro and cured lesions induced by *L. major* in BALB/c mice (15). The safety of SinaAmpholeish, and its vehicle (as control), produced in identical 15-g tubes by Razak Laboratories Company, Karaj, Iran, was first tested in animal model through using Draize and eye irritation test (16).

The objective of the current study was to evaluate the safety of SinaAmpholeish in a double-blind, randomized, phase 1 clinical trial in healthy human volunteers.

## Materials and Methods

### Study site and Ethical consideration

The study was carried out in DermaLab of Center for Research and Training in Skin Diseases and Leprosy (CRTSDL), Tehran University of Medical Sciences (TUMS), Tehran, Iran from 13 Jun to 13 Jul 2012.

The study was approved by Institutional Ethical Committee of CRTSDL with reference number J/423/204 and IRCT reference number IRCT20081130001475N12.

### Study product

Topical Nano-liposomal amphotericin-B 0.4% was prepared in 15-g tubes in a GMP condition at Razak Laboratories Company, Karaj, Iran, placebo was prepared identical to SinaAmpholeish, the placebo was similar to SinaAmpholeish except lack of Amphotericin-B, in another word placebo was empty liposomes.

### Study design

This study was a randomized, double blind, right-left, placebo-controlled, phase 1 safety clinical trial. The volunteers were selected from healthy individuals with the following eligibility criteria: male or female, aged 18–65 yr, signed an informed consent form. Those suffering from any skin diseases, had allergy, history of photosensitivity, use of systemic or topical anti-inflammatory drugs (including corticosteroids, antihistamines, etc.) within 1 month prior to the initiation of the trial were not included.

The study was conducted in two steps:

Step 1: Seven healthy volunteers were included in this step and were instructed to apply pea-sized (approximately 4 mg/cm^2^) of the SinaAmpholeish on a surface of 5×5 cm on volar surface of right or left forearms, 10 cm below elbow line, twice a day for one week without occlusion. The volunteers were instructed not to wash the product for at least 3 h after application and return the tubes after the end of study to check for compliance.
Step 2: Twenty volunteers were recruited in this step and instructed the same as step 1, except that SinaAmpholeish and its vehicle were used 3 times a day for 2 wk.

### Randomization and Blinding

Nano-liposomal formulation of amphotericin-B and its vehicle were prepared in identical tubes, labeled as 1 to 7, right or left according to a computer-generated randomization list, by a biostatistician outside the study team, and delivered to the study site. Neither the volunteers nor the investigators were aware of the type of medication applied on each side.

### Assessment

Any signs or symptoms of skin reactions (including pruritus, burning, skin redness, edema, and scaling) were recorded on a 0–3 scale:

0 = none
1 = mild (transient with no change in study protocol),
2 = moderate (persistent causing temporary discontinuation of application),
3 = severe (persistent causing complete discontinuation of application).

Before and after the end of application (1 wk in step 1 and 2 wk in step 2) skin temperature, pH, hydration, trans-epidermal water loss (TEWL), pigmentation, erythema, and sebum were measured on both application sites, after 30 min of acclimatization to temperature of 22 ± 3 ºC and humidity of 40 ± 5%, in DermaLab, Center for Research & Training in Skin Diseases & Leprosy, Tehran University of Medical Sciences. Measurements were done at 8–12 AM by corresponding probes of Multi Probe Adapter (MPA) system (Courage + Khazaka Electronic GmbH, Cologne, Germany). Volunteers were instructed not to wash the skin or apply any topical products on the day of measurement.

### Statistical analysis

The means and standard deviations of measured parameters before and after application of SinaAmpholeish and its vehicle were compared in both steps. Moreover, the change of each parameter (parameter after application *vs.* parameter before application) was compared between the two treatment sides in step 2. The frequency of skin reactions was also compared between the two application sides. The level of significance of differences was calculated with two-tailed paired t-test for means of dependent variables and Fisher’s exact test for proportions, and *P*<0.05 was considered as significant.

### Quality control/Quality assurance

The study was designed, conducted and reported according to the ICH/GCP guidelines (17).

## Results

### Step 1:

Seven adult volunteers were recruited and no skin reactions were observed after one week of application. The biophysical characteristics of skin before and after one week after application also showed no significant difference in hydration, TEWL, melanin, erythema, temperature, sebum and pH between SinaAmpholeish and its vehicle (*P*>0.05, Table 1).

**Table 1:** The biophysical characteristics of skin before and after 1 week of application of topical Nano-liposomal amphotericin-B 0.4% or its vehicle in 7 healthy volunteers

***Code***	***Maan***						
	***Hydration***	***TEWL***	***Melsnin***	***Erythema***	***Temperature***	***Sebum***	***pH***
A.F Before Rr	37.3	15.3	216.3	241.3	30.1	2	4.19
A.F.Before Lr	47.6	11.9	205.0	231.0	30.9	1	4.44
A.F After Rr	39.9	15.6	207.6	194.6	30.8	0	4.41
A.F.Before Lr	26.2	10.6	213.6	242.6	30.8	0	4.25
A.F.Before Rr	50.7	15.7	210.0	255.6	29.2	2	4.67
A.K Before Rr	50. 7	15.7	210.0	255.6	29.2	2	4.67
A.K Before Lr	40.2	7.4	204.3	238.3	29.4	1	4.30
A.K Before Rr	34.4	47.8	222.0	222.6	29.4	0	4.67
A.K Before Lr	22.6	25.8	193.6	261.3	29.0	0	4.99
A.M.Before Rr	42.4	22.2	220.6	221.6	32.2	0	5.37
A.M.Before Lr	34.5	11.1	195.3	199.6	32.0	0	5.22
A.M.Before Rr	32.3	11.1	226.6	288.3	31.6	0	5.64
A.M.Before Lr	27.8	5.7	197.0	232.0	30.7	0	5.42
E.J.Before Rr	33.4	13.9	318.6	239.0	30.9	1	4.36
E.J.Before Lr	27.6	11.4	278.3	295.6	30.8	1	4.43
E.J.Before Rr	36.8	14.2	316.6	357.3	31.3	3	4.30
E.J.Before Lr	36.0	13.1	301.0	322.0	29.5	1	4.50

### Step 2:

**Fig. 1:**
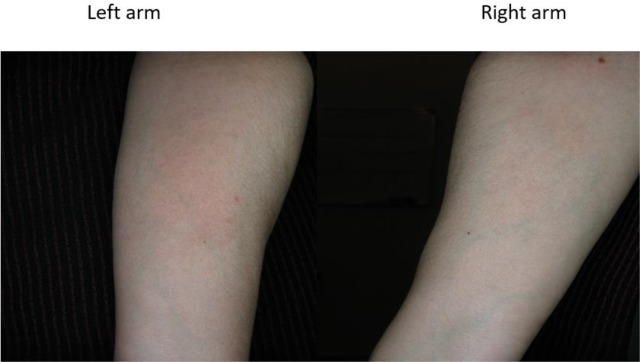
Severe skin reactions on both arms at day 4 of application of SinaAmpholeish, patient No.1

**Fig. 2:**
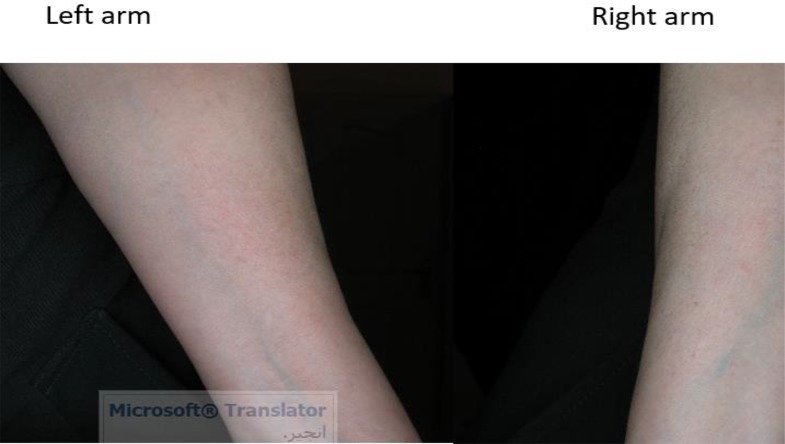
Severe skin reactions on both arms at day 4 of application of SinaAmpholeish, patient No.2

Twenty volunteers (F=12, M=8), with mean age of 37.8 ±10.3 yr (range, 24–61) were recruited in this step of the study and applied SinaAmpholeish or its vehicle 3 times a day for 2 wk. Two female volunteers aged 45 and 46 yr were withdrawn after 4 days of application due to severe skin reactions on both sides (Figs. 1 and 2). Therefore, 18 volunteers completed the trial according to the protocol.

The overall frequency and severity of local skin reactions at the site of application of SinaAmpholeish or its vehicle are shown in Table 2, and there was no significant statistical difference between the SinaAmpholeish and its vehicle. All of the reactions were mild and transient.

The mean and standard deviation of skin biophysical parameters; measured before and after 2 weeks of treatment are shown in Table 3. Moreover, the changes of each parameter from baseline (parameter after application – parameter before application) are shown in Table 4.

**Table 2:** The frequency and severity of local skin reactions at the site of application of 0.4% amphotericin-B liposomal preparation and its vehicle (n=18)

***Variable***	***Pruritus (%)***	***Burning***	***Edema***	***Erythema***	***Scaling***
0.4% Amphotericin-B liposomal preparation	9 (50)	7 (38.5)	7 (38.5)	7 (38.5)	0
Vehicle	9 (50)	5 (27.5)	5 (27.5)	5 (27.5)	0

**Table 3:** The means (standard deviations) of skin biophysical parameters measured before and 2 weeks after application of 0.4% amphotericin-B liposomal preparation (n=18)

***0.4% amphotericin-B liposomal preparation***	***Before application***	***After application***	***P-value (paired t-test)***
Temperature (°C)	31.9 (1.0)	32.4 (0.5)	0.09
TEWL (g/m2/hr)	9.9 (6.4)	13.8 (6.9)	0.123
Hydration (AU)	43.2 (11.4)	36.5 (14.8)	0.060
Surface lipid (micg/cm2)	7.6 (8.2)	2.5 (6.2)	0.057
Erythema index (AU)	202.28 (59.03)	208.75 (51.27)	0.743
Melanin index (AU)	207.75 (134.96)	153.33 (35.39)	0.100
pH	5.04 (0.59)	5.09 (0.78)	0.772

**Table 4:** The change in means (standard deviation) of skin biophysical parameters from baseline (parameter after application – parameter before application) after 2 weeks of application of 0.4% amphotericin-B liposomal preparation and its vehicle (n=18)

***Variable***	***0/4% amphotericin-B liposomal preparation***	***Placebo***	***P-value (paired t-test)***
Temperature (C)	0.44 (1.05)	0.41 (1.21)	0.798
TEWL (g/m2/hr)	3.88 (10.13)	6.34 (12.81)	0.149
Hydration (AU)	− 6.67 (13.91)	− 10.34 (18.09)	0.227
Surface lipid (micg/cm2)	− 5.12 (10.29)	− 7.00 (13.69)	0.529
Erythema index (AU)	6.47 (82.42)	40.14 (264.55)	0.598
Melanin index (AU)	− 54.42 (132.60)	107.44 (173.07)	0.004
pH	0.06 (0.82)	5.31 (14.93)	0.154

## Discussion

Leishmaniasis is a parasitic neglected disease with various clinical manifestations including CL, currently no safe and efficacious treatment is available to treat CL. Standard WHO recommended treatment is antimoniate derivatives which has been in use for last 70 yr, this treatment needs multiple injections and accompanies side effects and the efficacy is not high (6–9, 13, 18).

Amphotericin B is effective against different *Leishmania* species but it is toxic (11, 12). Development of topical treatment for CL is a major step to alleviate the pain of treatment. A few topical ointments have been developed to treat CL but none is in the market now (9–13, 19–21).

A new topical Nano-liposomal formulation of amphotericin B has been developed (0.4% Amphotericin-B Nano-liposomal preparation). This formulation has been shown to be effective against *L. major* and *L. tropica in vitro* and was able to cure lesions induced by *L. major* in BALB/c mice (15). The safety evaluation is usually tested in animal model (22), the results of SinaAmpholeish in animal model were satisfactory (16).

The current study was designed to assess the safety of SinaAmpholeish in human healthy volunteers in a double-blind design study completed under the ICH/GCP guidelines (17). Safety and side effects of cosmetic and topical formulations are checked in human using different skin physical parameters (23, 24). In the current study, seven biophysical skin parameters including temperature, TEWL (transepidermal water loss), hydration, surface lipid, erythema index, melanin index, and pH were evaluated in standard conditions before and 2 weeks after application. The results showed no adverse effects when the SinaAmpholeish and its vehicle were used twice a day for seven days. However, when SinaAmpholeish and its vehicle were used 3 times a day for two weeks, both SinaAmpholeish and its vehicle showed transient severe local skin reactions in 2 of 20 volunteers (10%) after application of Nano-liposomal Amphotericin-B 0.4% or its vehicle three times a day for 2 weeks. Mild transient reactions were also seen in half of the remaining subjects. As the reactions to the vehicle were similar to the reaction to the formulation, it is implied that the reactions are due to the composition of the vehicle, and not the active drug.

Based on the results of animal model studies and the current results it is worth to check further the formulation in a phase 2 trial in human. Since there was no adverse effect with twice a day application, it was decided to use the SinaAmpholeish twice a day in phase 2 clinical trial.

## Conclusion

Topical nano-liposomal form of amphotericin B (SinaAmpholeish) using twice a day is safe. It could be used for further efficacy in human trials.

## Ethical considerations

The study was approved by Institutional Ethical Committee of CRTSDL with reference number J/423/204 and IRCT reference number IRCT20081130001475N12, every volunteer singed an informed consent form.

Ethical issues (Including plagiarism, informed consent, misconduct, data fabrication and/or falsification, double publication and/or submission, redundancy, etc.) have been completely observed by the authors.
